# Bioactive compounds in fermented foods: a systematic narrative review

**DOI:** 10.3389/fnut.2025.1625816

**Published:** 2025-07-02

**Authors:** İbrahim Ender Künili, Vildan Akdeniz, Aslı Akpınar, Şebnem Öztürkoğlu Budak, José Antonio Curiel, Mustafa Guzel, Cem Karagözlü, Muzeyyen Berkel Kasikci, Grech Perry Mario Caruana, Małgorzata Starowicz, Christèle Humblot, Erhan Keyvan, Christophe Chassard, Smilja Pracer, Guy Vergères, Harun Kesenkaş

**Affiliations:** ^1^Department of Fishing and Fish Processing Technology, Faculty of Marine Sciences and Technology, Çanakkale Onsekiz Mart University, Çanakkale, Türkiye; ^2^Department of Dairy Technology, Faculty of Agriculture, Ege University, Izmir, Türkiye; ^3^Department of Food Engineering, Faculty of Engineering and Natural Science, Manisa Celal Bayar University, Manisa, Türkiye; ^4^Department of Dairy Technology, Faculty of Agriculture, Ankara University, Ankara, Türkiye; ^5^Department of Food Technology, National Institute of Agricultural and Food Research and Technology (INIA-CSIC), Madrid, Spain; ^6^Department of Food Engineering, Hitit University, Corum, Türkiye; ^7^Department of Food Sciences and Nutrition, Faculty of Health Sciences, University of Malta, Msida, Malta; ^8^Department of Chemistry and Biodynamics of Food, Institute of Animal Reproduction and Food Research of the Polish Academy of Sciences, Olsztyn, Poland; ^9^French National Research Institute for Sustainable Development (IRD), Montpellier, France; ^10^Department of Food Hygiene and Technology, Faculty of Veterinary Medicine, Burdur Mehmet Akif Ersoy University, Burdur, Türkiye; ^11^Unité Mixte de recherche sur le Fromage, INRAE, UCA, VetAgro Sup, Aurillac, France; ^12^Institute for Biological Research Sinisa Stankovic, National Institute of Republic of Serbia, University of Belgrade, Belgrade, Serbia; ^13^Research Division Microbial Food Systems, Agroscope, Berne, Switzerland

**Keywords:** fermented foods, bioactive compounds, fermentation, health benefits, clinical effects, clinical trials

## Abstract

This systematic narrative review aims to catalogue bioactive compounds that are formed during the fermentation process and identified as contributors to positive health outcomes in human clinical studies where fermented foods were used as dietary interventions. Under “The European Cooperation in Science and Technology” (COST) Action CA20128 – “Promoting Innovation of Fermented Foods” PIMENTO framework, a systematic literature search was conducted across PubMed, Scopus, and the Cochrane Library. The search initially identified 2,411 studies, of which 50 met the predefined inclusion criteria. In all included studies, fermented foods were used as nutritional interventions, and the bioactive compounds they contained were either hypothesised or directly analysed in relation to statistically significant health effects observed in humans. Findings were summarised in “Population, Intervention, Comparator, Outcome” (PICO)-structured tables and classified by health domain. The reported effects were grouped under the following categories: cardiovascular health, lipid metabolism, glucose regulation, immune modulation, neuroprotection, liver function, and other health outcomes. Across the included studies, a total of 31 bioactive compounds and/or compound groups were identified in fermented foods, including bioactive peptides, polyphenols (epicatechin, genistein), *γ*-aminobutyric acid (GABA), acetic acid, curcumin, and arabinoxylans. The mechanisms of action associated with these compounds were explored in the non-systematic section of the review, supported by findings from *in vitro*, animal and human studies. By linking individual bioactive compounds derived from fermented foods to clinical outcomes and underlying mechanisms, this review provides a comprehensive and functional resource for nutritional science, fermented food innovation, and public health applications.

**Systematic review registration:**

https://osf.io/jn8pf/.

## 1 Introduction

Fermented foods have long been appreciated for their extended shelf life, distinctive sensory qualities, and potential health benefits. Microbial fermentation enhances the nutritional and functional properties of food by producing a wide range of bioactive compounds, including peptides, polyphenols, organic acids, short-chain fatty acids (SCFAs), exopolysaccharides (EPS), and vitamins. These compounds have been associated with physiological effects such as improved cardiovascular function, lipid metabolism, glycaemic control, immune modulation, neuroprotection, and oxidative stress reduction—positioning fermented foods as valuable components of functional dietary strategies (1, 2).

Although evidence supporting the health benefits of fermented foods has grown, a range of issues complicates the interpretation of clinical findings and limits the ability to infer causality. In particular, the variability in study designs, intervention protocols, and outcome measures, combined with the interindividual variability of the metabolic response to food intake, continues to hinder the comparability and generalisability of findings. Also, the variability in food matrices, fermentation processes, and microbial strains remains a significant challenge. Furthermore, while numerous bioactives have been identified, a systematic classification linking specific compounds to clinical outcomes in humans is still lacking.

This review addresses these gaps by systematically evaluating human interventional and observational studies, reporting a statistically significant clinical outcome, in which fermentation-derived compounds were discussed as potential mediators of the bioactivity of fermented foods. A central feature of this review is its compound-centric approach, which departs from traditional models that group findings by food type or health condition. Instead, each bioactive compound is mapped to its source fermented food and evaluated based on clinical outcomes.

The review is guided by the hypothesis that fermentation can generate or enhance compounds capable of producing measurable health effects in clinical settings. By identifying and cataloguing these compounds, the study aims to clarify their functional roles and provide a scientific basis for their use in food and health applications. The central research question—formulated within the framework of the COST Action CA20128 “Promoting Innovation of Fermented Foods” (PIMENTO), specifically Working Group 3 (WG3)—is: *Which compounds generated through food fermentation are associated with clinically relevant outcomes in human studies?* This is addressed through a structured analysis of clinical trials, resulting in a comprehensive overview of bioactive compounds and their health impacts (3).

To complement the systematic component, the review also includes a narrative synthesis of mechanisms of action, grouped by health outcomes and based on evidence from *in vitro,* animal and human studies. By integrating clinical findings with mechanistic insights, the review provides a compound-level understanding of how fermentation-derived bioactives contribute to health. This approach offers valuable perspectives for fermented food technology, nutritional science.

## 2 Methods

### 2.1 Study design and protocol

This study followed a review-specific protocol adapted from the general framework developed by PIMENTO Working Group 3 (WG3) (3). The protocol integrates a systematic review of human clinical studies with a non-systematic assessment of underlying mechanisms of action.

The systematic review process adhered to the methodological steps proposed by Muka et al. (4), was structured in accordance with the PROSPERO format, and aligned with the indicative guidance of the European Food Safety Authority (EFSA), particularly regarding the evaluation of mechanisms of action. The protocol was initially developed as a project^1^ and subsequently registered on the Open Science Framework (OSF) (5).

### 2.2 Literature search

A systematic literature search was conducted across PubMed, Scopus, and the Cochrane Library, covering studies published between 1 January 1970 and 31 August 2023. An additional search, extending from August 2023 to December 2024, was performed to identify potentially relevant studies and screened using the same methodology as in the initial phase. Only publications written in English were considered for inclusion.

The search strategy was based on the PIMENTO WG3 Study Protocol, as outlined in the position paper by Todorovic et al. (3), and was further adapted for this review using Study Protocol-Satellite 1 (S1) (version 3.10.2024). The generic search strings, developed for all 16 systematic reviews conducted within the PIMENTO framework—covering a wide range of fermented foods, human clinical study types, and dietary intake assessments—were applied.

The initial literature search and title/abstract screening were performed using an inclusive search string to capture all fermented food-related studies. Review-specific criteria were then applied during the secondary title/abstract screening and initial full-text screening to ensure the inclusion of studies relevant to this review’s focus.

All retrieved human studies were systematically screened, and eligible publications included human intervention (efficacy) studies such as randomised controlled trials, non-randomised controlled trials, and other clinical intervention studies. Observational studies, including cohort, case–control, and cross-sectional studies, were also considered. Systematic reviews, with or without meta-analyses, were screened to identify any additional relevant studies. Animal and *in vitro* studies were excluded from this review.

### 2.3 Study selection criteria

#### 2.3.1 Inclusion and exclusion criteria

##### 2.3.1.1 Population

Inclusion criteria: human adults aged 18–70 years.

##### 2.3.1.2 Intervention/exposure

Inclusion criteria: consumption of fermented foods listed in the PIMENTO search strings, including dairy, meat, fish, fruits, vegetables, beverages, legumes, cereals, and grains.

Exclusion criteria: non-nutritional applications (e.g., nasal or topical use). Studies with probiotics, unless introduced at fermentation onset and contributing to the fermentation process. Studies including confounding factors in tested food, such as prebiotic fibres or added bioactive compounds. All studies investigating coffee for which the contribution of fermentation to the large amount of bioactive compounds is unknown or contribution to health outcome may generally not directly related to a specific compound or compound group. Studies investigating tea, which is not or rarely fermented.

##### 2.3.1.3 Comparators

Inclusion criteria: comparators included the absence of fermented food consumption, lower intake frequency, non-fermented counterparts, or placebo treatments.

##### 2.3.1.4 Outcomes

Inclusion criteria: any statistically significant primary or secondary clinical endpoint from human studies meeting the inclusion criteria for population, intervention/exposure, and comparator for which the bioactivity of a compound, produced or modulated by fermentation, has been postulated (i.e., presented in the title and/or the abstract or introduction of the published article).

Exclusion criteria: studies in which the bioactive compound presumably mediating the effect of the fermented food on the clinical outcome is not produced or modulated by the fermentation process.

### 2.4 Study selection and data extraction

#### 2.4.1 Selection and screening

Relevant studies identified across the databases were exported and processed using the CADIMA software for duplicate removal (6). During the title and abstract screening phase, studies were excluded if they did not meet the criteria for population, intervention, comparator or clinically measured health outcomes.

In the full-text screening stage, review-specific criteria were applied: (i) intervention—studies in which bioactive compounds were postulated in the title, abstract, or hypothesised in the introduction, either with direct results or through citations of prior studies on the hypothesised compounds; and (ii) outcome—studies that reported statistically significant health effects across any clinically relevant health endpoint.

At each screening stage—including consistency checks during title, abstract, and full-text review to evaluate the effectiveness of the S1-specific protocol in CADIMA—two independent reviewers assessed the studies. Discrepancies were resolved by a third reviewer through consensus.

#### 2.4.2 Data extraction and documentation

The S1-specific data extraction form, a modified version of the PIMENTO WG3 data extraction form based on *Cochrane’s Conducting an Intervention Review* handbook (7), *EFSA Appendix B* for human efficacy studies (8), and *STROBE* guidelines for observational studies (9) were used as guidance for preparation of the data extraction form for the included studies.

### 2.5 Non-systematic review components

This review documented clinical indications investigated for the identified bioactive compounds in fermented foods in accordance with the review-specific study protocol. Additional supportive evidence related to the health-promoting effects of these bioactive compounds and their mechanism of action on health-promoting activity was documented with supportive evidence identified from the literature.

### 2.6 Summary of findings

This review collects reports from human clinical studies investigating the health effects of bioactive compounds present in fermented foods as intervention/exposure. Only studies were included if the bioactive compound either increased during fermentation from the raw matrix or formed entirely as a result of the fermentation process. In addition, the compound had to be postulated or identified within the fermented food, and the study had to report statistically significant clinical outcomes. The selection and interpretation of the reports were guided by Section 5 of the EFSA guidance (8), with particular emphasis on Sections 5.2.1 and 5.2.2, which served as indicative references to ensure methodological consistency throughout the review. Mechanistic explanations for the observed health effects were considered when described in the original clinical studies. Additionally, this was supported by additional human, animal, or *in vitro* studies involving the same compound, in line with the principles outlined in Section 5.2.3 of the EFSA guidance. This review did not include detailed food characterisation nor categorise the strength of evidence according to predefined EFSA evidence statements. Instead, its primary objective was to identify and summarise clinical studies that reported statistically significant associations between the consumption of fermented foods containing specific bioactive compounds and defined health outcomes. Accordingly, studies were excluded even if they met the PICO criteria, under the following conditions:

- The reported health outcomes associated with the consumption of fermented foods containing postulated bioactive compounds were not statistically significant compared to control or comparator groups; or.
- The study did not refer to, hypothesise about, or postulate the role of any specific bioactive compound.
- The selection of clinical studies presented in this review is biassed in that it purposely presents clinical studies with significant outcomes. Other reviews of the PIMENTO series (3) take an unbiased approach by conducting a systematic analysis of the evidence for the impact of fermented foods on selected clinical outcomes.

## 3 Results and discussion

### 3.1 Literature search results

**Figure 1:**
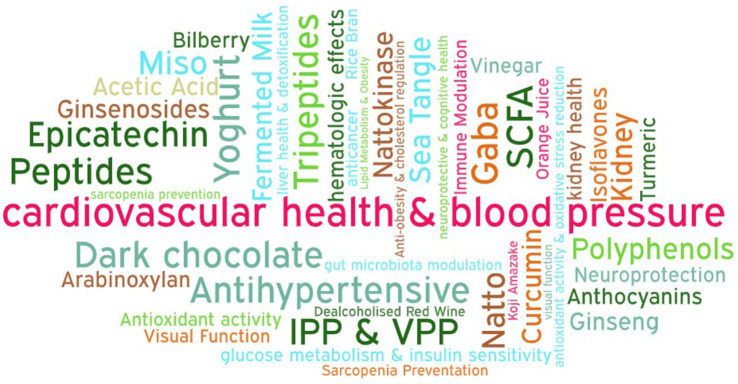
PRISMA flow diagram illustrating the study selection process and results of the systematic literature search.

The screening process began with the identification of 2,411 publications (Figure 1). In the initial phase, 438 duplicate records were removed. A further 1,649 studies were excluded following title and abstract screening. This resulted in 324 studies deemed potentially relevant, which underwent full-text screening based on additional review-specific inclusion criteria. Following full-text assessment, 294 studies were excluded for failing to meet these criteria.

An additional 63 potentially relevant studies were identified through the reference lists of systematic reviews encountered during the screening process, and screening and selection criteria were applied to all potential studies which were not encountered during the initial literature search. Ultimately, from initial, secondary and additional literature searches, 50 papers were included in the final synthesis. These covered a diverse range of fermented foods—including dairy products, cereals, grains, vegetables, and beverages—and were conducted across varied populations and clinical endpoints.

### 3.2 Overview of the included studies

The included 50 publications, each reporting one different human clinical study (1 observational study and 49 intervention studies), were categorised according to the predominant health benefit reported and are summarised in Table 1. Also, Supplementary Table 1 provides additional data for bioactive compounds in fermented foods, including health benefits, fermentation parameters, raw food sources, and types of fermenting microorganisms.

**Table 1:** Bioactive compounds in fermented foods evaluated in clinical studies designed according to PICO criteria.

Type of health benefits	Population	Intervention/Exposure		Comparator (Control/placebo)	Outcome		References
		Bioactive compound(s)	Fermented food		Primary outcome and secondary outcome (if any)	Significant change (↓↑)	
Cardiovascular health and blood pressure regulation	24 females, 20 males (mean age 63.55)	Polyphenols (n.s)	Dark chocolate 6.3 g/day	Polyphenol-free white chocolate	BP reduction	SBP ↓2.9 mmHg, DBP ↓1.9 mmHg, hypertension prevalence ↓86% → 68%	(20)
	34 men and 60 women hypertensive adults	IPP and VPP peptides	Fermented milk 150 mLx2/day	Control product	BP reduction	SBP ↓6.7 mmHg, DBP ↓4.1 mmHg	(10)
	39 hypertensive volunteers aged 30.2–61.7 years	IPP and VPP peptides	Fermented milk 150 mL/day	Control product	BP reduction	SBP ↓ 6.7 mmHg, DBP ↓ 3.6 mmHg	(11)
	67 men at high cardiovascular risk (mean age 60)	Polyphenols (n.s)	Dealcoholized red wine 272 mL/day	Baseline	BP reduction	SBP ↓ 5.8 mmHg, DBP ↓ 2.3 mmHg	(41)
	50 prehypertensive adults aged 30–65 years	10–15 kDa bioactive peptides	Fermented goat milk tablets 1.25 g × 6/day	Placebo tablets	BP Reduction	SBP ↓ 12.5%, DBP ↓ 9.3%	(12)
	80 Adults with high-normal BP/mild hypertension	IPP and VPP peptides	Fermented milk tablets 6 tablets/day	Placebo tablets	BP Reduction	High-normal BP: SBP ↓ 3%, DBP ↓ 7%, Mild hypertension: SBP ↓ 7%, DBP ↓ 8%	(13)
	42 Men aged 23–59 years with borderline hypertension	IPP and VPP peptides	Fermented Sour Milk 160 g/day	Artificially acidified milk drink	BP Reduction	SBP ↓ 5.2 mmHg, DBP ↓ 2.0 mmHg	(14)
	40 adults with mild hypertension	IPP and VPP peptides	Sour Milk 1.5dL/day	Regular sour milk	BP Reduction	SBP ↓ 16 mmHg (active) vs. ↓ 14 mmHg (placebo), Overall ↓ 2.6 mmHg greater SBP reduction with active product	(15)
	16 Men aged 25–32 years with 19–23 BMI	Epicatechin	Cocoa drink with high (917 mg)/low (37 mg) flavanol	Baseline	Improved vascular function	PAT index: High flavanol ↑ 68.9%, Low flavanol ↑ 18.1%	(16)
	79 adults with elevated BP (mean age 31.11)	Nattokinase	Fermented soy extract Natto 100 mg nattokinase/day	Placebo capsules	BP reduction	DBP ↓, men DBP ↓, vWF ↓ in women, renin activity ↑ 66% (test) vs. ↑ 8% (placebo)	(28)
	38 adults aged 40–69 years with high-normal BP/stage I hypertension	=3 kDa Peptides	Miso 32 g with 3.8 g salt/day	Soy food without salt	BP reduction	Nighttime SBP ↓ 9.4%, DBP ↓ 8.0%	(29)
	70 adults aged 39–61 years with mild hypertension	IPP and VPP peptides	Liquid yoghurt 150 g twice/day	Placebo drink	BP reduction	SBP ↓ 8.4 mmHg (week 1), ↓ 13.9 mmHg (week 8), DBP ↓ 6.0 mmHg (week 1), ↓ 9.1 mmHg (week 8)	(17)
	60 adults aged 18–24 years	Flavanols (epicatechin and procyanidin)	Dark chocolate 10 g/day (>75% cocoa)	No concomitant intervention	Improved vascular function	FMD ↑ 9.31%, ASI ↓ 0.16–0.13, PWV ↓ 6.13–5.83	(21)
	89 hypertensive adults aged 25–55 years	IPP and VPP peptides	Fermented milk 200 mL/day	Placebo drink	Reduced arterial stiffness	Augmentation index ↓ 1.53%	(18)
	106 adults with high-normal BP (mean age 38.3)	IPP and VPP peptides	Liquid yoghurt 150 g twice/day	Placebo drink	BP reduction	SBP ↓ 5.5 mmHg (week 6), ↓ 6.1 mmHg (week 12), DBP ↓ 3.6 mmHg (week 6), ↓ 3.8 mmHg (week 12)	(19)
Lipid metabolism, cholesterol homeostasis and obesity management	9 healthy adults aged 21–25 years	Flavonoids (n.s)	Fermented orange juice 500 mL	Orange juice	Hypolipidemic	LDL-C increase lower (621.9 vs. 718.6), apo-B ↓409.7	(56)
	76 Hypercholesterolemic adults aged 35–60 years	Acetic acid	Red date vinegar 30 mL/day	Placebo	Anti-hypercholesterolemic effects	Total cholesterol ↓ 19.80% (197.6 g), LDL ↓ 34.90% (109.8 g), TG ↓ 5.34% (161.2), HDL ↑ 5.75% (44.1 g)	(53)
	30 healthy volunteers (mean age 33.9)	Polyphenols (n.s.)	Fermented orange juice 500 mL/day	Habitual diet	Increased antioxidant status, reduced lipid peroxidation	ORAC ↑43.9%, uric acid ↓8.9%, TBARS ↓30.2%	(57)
	9 adult males aged 45–50 with no chronic disease	Polyphenols (n.s)	Dealcoholized red wine 272 mL/day	Baseline	Anti-obesity and cholesterol regulation	Fecal concentration of *Bifidobacterium* ↑, Urinary phenolic metabolites ↑	(58)
	73 healthy adults aged 20–50 years	Epicatechin, dihydrophenyl derivatives	Dark chocolate 25 g twice daily	Controlled diet	Increased HDL and lipid metabolism improvement	HDL ↑6%	(22)
	60 adults aged 19–65 years with BMI > 23	Isoflavones (genistein, daidzein), small peptides	Doenjang 9.8 g/day	Placebo pills	Anti-obesity	Catalase activity ↑ 2x	(30)
	56 adults aged 23–71 years	Soluble fibre (β-glucan)	Fermented ropy oat-based products 200 mLx3/day	Fermented dairy-based product	Total cholesterol reduction	Total cholesterol ↓ 6%	(66)
	161 hypercholesterolemic adults with mean 26.3 BMI (mean age 54.5)	Lactoferrin, immunoglobulin, exopolysaccharides	Malleable Protein Matrix (MPM) 2×15/day	Gelatin-based placebo	Triglyceride reduction	Triglycerides ↓ 9.8%	(68)
	41 women with hyperlipidaemia	Dietary fibre	Tempeh Gembus 206 g/day	Control	LDL-C reduction	LDL-C means difference ↓24.14 mg/dL	(31)
	53 overweight/obese adults aged 19–65 years	Capsaicin	Kochujang 32 g/day	Placebo pills	Reduced TG, TG/HDL ratio, dietary sodium, improved insulin sensitivity	TG ↓ 14.1%, TG/HDL ↓ 13.6%, insulin sensitivity ↑	(32)
	30 healthy adults aged 19–55 years	Capsaicin, daidzein, genistein	Kochujang 34.5 g/day	Baseline	Total cholesterol reduction	TC ↓ 9.75%	(33)
	21 normal weight 17 obese adults aged 18–45 years	Phenolic compounds (n.s)	Kombucha 200 mL/day	Baseline	Improved gut microbiota	*Subdoligranulum* ↑ in obese group	(74)
	55 healthy adults aged 26–68 years with TG 120–200 mg/dL	Monacolin K, dimerumic acid	*Monascus pilosus* garlic extract capsules (MGFE) 900 mg/day	Baseline	Reduced TG, cholesterol, LDL/HDL	TG ↓ 14.9%, total cholesterol ↓ 8.6%, LDL ↓ 14.2%	(72)
Antioxidant activity and oxidative stress reduction	20 healthy adults aged 20–56 years	Epicatechin	Procyanidin-rich chocolate (27 g, 53 g, 80 g)	Control	Increased plasma epicatechin, reduced lipid oxidation	Plasma epicatechin: ↑ 133% (27 g), ↑ 258% (53 g), ↑ 355% (80 g), TBARS ↓ 20% (80 g), Antioxidant capacity ↑ 253 s	(23)
	21 healthy adults aged 18–50 years with mean 21.6 BMI	Epicatechin	Cocoa Beverage 250 mL	Milk	Increased antioxidant activity	Total antioxidant activity ↑ 62.35%	(24)
	48 healthy males aged 25–60 years with high γ-GT	γ-aminobutyric acid (GABA)	Fermented Sea Tangle 6 capsules/day	Placebo	Reduced serum γ-GT, MDA, increased SOD, CAT	γ-GT ↓ 23.51 U/L, SOD ↑ 49.60 U/mL, CAT ↑ 13.64 mU/mL, MDA ↓ 10.47 qmol/mg	(78)
	101 neo-diabetic adults aged 25–60 years	Polyphenols (n.s)	Fermented Papaya Preparation (FPP) 1 sachet x2/day	Water	Improved CRP, LDL/HDL, uric acid, antioxidant status	CRP ↓ 13.3%, LDL/HDL ↓ 2.6%, uric acid ↓	(75)
Glucose metabolism and insulin sensitivity	16 healthy individuals aged 20–50 years with 18–25 BMI	Insoluble dietary fibre, organic acids (lactic and acetic acid)	Sourdough Bread	Commercial par-baked wheat bread	Lower glycemic index	Glycemic AUC reduction by 8%, insulin AUC reduction by ~26%	(80)
	16 male cyclists aged 19–38 years	Epicatechin, theobromine	Dark Chocolate (70% cocoa)	Control (cocoa-depleted)	Increased plasma glucose, insulin, muscle glycogen utilisation, reduced plasma glucose oxidation	Plasma glucose ↑ 8%, glucose oxidation ↓ 18%, glycogen utilisation ↑ 15%	(25)
	14 healthy adults aged 21–28 years	Caffeic, ferulic, and sinapic acids	Rye breads from five different rye varieties	White wheat bread	Glycemic regulation	Amilo/Rekrut rye bread ↓ insulin indices	(85)
	30 men aged 20–70 years and postmenopausal women aged =70 years	γ-polyglutamic acid (γ-PGA)	High-γ-PGA Natto 40 g/meal	Low-γ-PGA Natto	Postprandial glucose reduction, dysglycemia prevention	IAUC glucose ↓ 0-15 min and 0-30 min, insulin ↓ 0-45 min	(34)
Hematologic effects	18 healthy men (mean age 36)	Epicatechin	Dark Chocolate (90% cocoa)	Baseline	Platelet inhibition	Positive correlation between flavanol metabolites and platelet aggregation	(26)
	42 healthy adults aged 23–65 years	Flavan-3-ol, catechin	Flavan-3-ol enriched dark chocolate (60 g)	Standard dark chocolate	Platelet function, atherogenesis	↓ ADP-induced platelet aggregation	(27)
	24 athletes (mean age 19.6)	Bioactive peptides	High-Protein Concentrated Pro-Yogurt (3 times per day/5 days per week)	Plain control yogurt	Increased haemoglobin levels	Haemoglobin: ↑ 1.15 g/dl (group 1), ↑ 1.8 g/dl (group 2)	(42)
	12 healthy males with mean 21.6 BMI (mean age 22.3)	Nattokinase	Natto-derived natokinase (2000 FU/ capluse)	Placebo gel capluse	Enhanced fibrinolysis, anticoagulation	D-dimer ↑ 44.5% (6 h), FDP ↑ 21.2% (4 h), aPTT prolonged	(35)
Immune modulation and inflammation reduction	10 healthy Italian adults aged 30–65 years	Polyunsaturated fatty acids (CLA)	Pecorino cheese 200 g/week	Placebo (cow cheese)	Reduction in inflammatory markers, TNF-α	IL-6 ↓ 43%, IL-8 ↓ 36%, TNF-α ↓ 36%	(100)
	80 adults aged 25–70 years with 22.74–23.54 BMI and 4,000–8,000 cells/μL WBC count	Arabinoxylan	Fermented rice bran (RBEP)	Placebo group (cornstarch-based)	Enhanced immune activation	Increased IFN-γ levels	(105)
Neuroprotective and cognitive health effects	17 healthy females (mean age 40.9)	GABA	Non-alcoholic beer 333 mL/day	Control (No beer consumption)	Improved sleep latency and reduced stress	Sleep latency ↓40%, total activity ↓27%, state anxiety ↓12.5%	(111)
Liver health and detoxification	60 adults aged 20–70 years with >40 IU/L ALT	Curcumin	Fermented Turmeric Powder (3 g × 2 capsules × 3 times/day)	Placebo capsules	Reduced ALT, AST	ALT ↓ 26.5%, AST ↓ 23.1%	(116)
	90 adults aged 19–70 years with elevated ALT	Ginsenosides (Compound K)	Fermented ginseng powder low-dose (125 mg/day) or high-dose (500 mg/day)	Placebo tablets	Reduced GGT, hs-CRP, fatigue improvement	GGT ↓ 13.5 IU/L, hs-CRP ↓ 1.51 mg/L (low-dose group), decreased MFS score (high-dose group)	(118)
Others	18 males, 26 females aged 20–66 years	Oligosaccharides and Glycosylceramides	Koji Amazake (118 g/day)	Rice syrup	Increased defecation frequency and changes in gut microbiota	Defecation frequency ↑ (5.41 vs. 4.18 days), fecal weight ↑ (724 g vs. 501 g), Blautia ↓, Bacteroides ↑	(121)
	72 subjects with nephrolithiasis history	Acetic acid	Dietary vinegar (5 mL × 3/day)	Control	Prevention of CaOx nephrolithiasis	↑ increased citrate and ↓ reduced calciumin urinary excretion	(124)
	18 prostate cancer patients (mean age 69.81)	Enterolactone, enterodiol, matairesinol	Rye bran bread (155 g/day)	Placebo bread (135 g/day)	Anti-carcinogenic effects	Apoptotic rate ↑ TUNEL: 46.42%, Ki-67: 14.58%, p27: −3.42%	(126)
	192 men, 159 women patients with type 2 diabetes	Isoflavones (n.s)	Habitual miso consumption	Non-consumers	Lower prevalence of sarcopenia in women	Sarcopenia: 18.8% (Miso) vs. 42.3% (non-Miso)	(36)
	30 healthy volunteers aged 31–53 years with myopia	Anthocyanins (Cyanidin, Delphinidin, Malvidin, Peonidin, Petunidin)	Fermented Bilberry Extract (200 mg x2/day)	Placebo	Improved amplitude of accommodation and mesopic CS	Amplitude of accommodation ↑ (4.62–5.33 diopters), AULCSF ↑ (1.04–1.13)	(129)

ACE, Angiotensin-converting enzyme; ALT, Alanine aminotransferase; apo-B, Apolipoprotein B; ASI, Arterial stiffness index; AST, Aspartate aminotransferase; AULCSF, Area under the log contrast sensitivity function; BMI, Body mass index; BP, Blood pressure; CAT, Catalase; COX, Cyclooxygenase; CVD, Cardiovascular disease; FDP, Fibrin degradation products; FMD, Flow-mediated dilation; GABA, Gamma-aminobutyric acid; GGT, Gamma-glutamyl transferase; GI, Gastrointestinal; GM, Gut microbiota; HDL, High-density lipoprotein; hs-CRP, High-sensitivity C-reactive protein; IAUC, Incremental area under the curve; IFN-γ, Interferon-gamma; IL, Interleukin; K-MMSE, Korean Mini-Mental State Examination; Ki-67, Proliferation-associated nuclear protein; LDL, Low-density lipoprotein; MDA, Malondialdehyde; MFS, Multidimensional Fatigue Score; n.s., Not Specified; NO, Nitric oxide; PAT, Peripheral Arterial Tonometry index; p27, Cyclin-dependent kinase inhibitor p27^Kip1; PPAR, Peroxisome proliferator-activated receptor; PPG, Postprandial glucose; PPs, Polyphenols; PWV, Pulse wave velocity; ROS, Reactive oxygen species; SCFAs, Short-chain fatty acids; SOD, Superoxide dismutase; TBARS, Thiobarbituric acid reactive substances; TC, Total cholesterol; TG, Triglycerides; TNF-α, Tumour necrosis factor-alpha; TUNEL, Terminal deoxynucleotidyl transferase dUTP nick end labelling.

**Figure 2:**
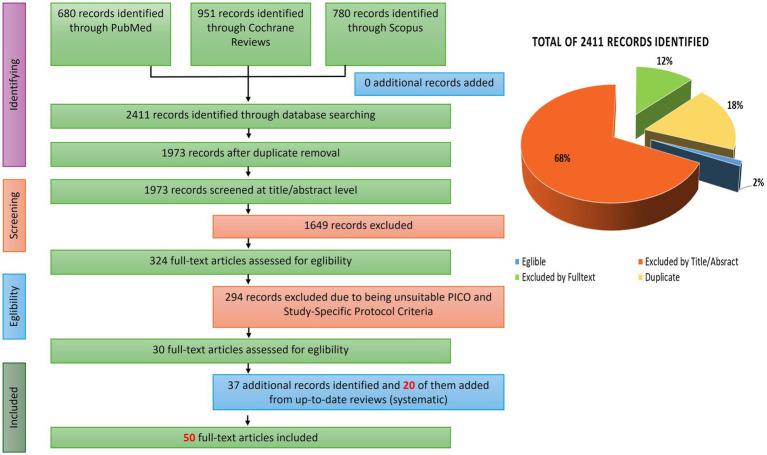
Word cloud illustrating the most frequently reported health benefits, bioactive compounds, and fermented foods identified in the included clinical studies.

The most frequently investigated health domain, bioactive compound and fermented food were summarised as a word cloud in Figure 2. The most studied health domain was cardiovascular health and blood pressure regulation (15 studies), followed by lipid metabolism, cholesterol homeostasis and obesity management (13 studies). Other categories included antioxidant activity and oxidative stress reduction, glucose metabolism and insulin sensitivity, and hematologic effects, each represented by 4 studies. Two studies each focused on neuroprotective and cognitive health effects and liver health and detoxification, while the remaining studies were grouped under “others,” covering diverse benefits such as gut microbiota modulation, kidney health, anticancer effects, sarcopenia prevention, and visual function enhancement.

In terms of intervention/exposure models, fermented dairy products (e.g., fermented milk and yoghurt) were the most studied (12 studies), primarily in the cardiovascular domain. These interventions focused on bioactive peptides—particularly tripeptides such as isoleucine-proline-proline (IPP), valine-proline-proline (VPP), as well as 10–15 kDa and =3 kDa peptide fractions—as the key bioactives responsible for angiotensin-converting enzyme (ACE) inhibition and blood pressure regulation (10–19).

The second most common intervention involved cocoa-based fermented products such as dark chocolate and cocoa beverages related bioactive compounds including flavanols, polyphenols, epicatechin and procyanidins. These compounds were investigated for their roles in improving vascular function, antioxidant capacity, lipid metabolism, and platelet aggregation (16, 20–27).

Fermented soy-based foods such as *natto*, *miso*, *doenjang*, *tempeh gembus*, and *kochujang* were also commonly studied. These products contain diverse bioactive compounds including nattokinase, small peptides, polyglutamic acid, isoflavones, and capsaicin, with effects reported across cardiovascular, lipid, metabolic, and inflammatory pathways (28–36). Other frequently studied fermented products included fermented orange juice, fermented sea tangle, fermented rice bran, fermented turmeric, fermented ginseng, kombucha, and *Monascus pilosus* garlic extract. These were investigated for a range of outcomes such as antioxidant capacity, anti-inflammatory effects, liver enzyme regulation, cognitive performance, and microbiota modulation.

Study populations were diverse in health status and demographics. The cardiovascular group largely consisted of middle-aged and older adults with prehypertension, hypertension, or elevated cardiovascular risk. In contrast, studies on lipid metabolism and obesity management included healthy individuals, overweight/obese subjects, or those with mild hypercholesterolemia. Many studies recruited generally healthy participants, while others specifically targeted populations with metabolic disorders, inflammation, or liver dysfunction.

## 4 Bioactive compounds associated with cardiovascular health and blood pressure regulation

Clinical studies have identified several bioactive compounds in fermented foods with demonstrated cardiovascular benefits, including ACE-inhibitory peptides (Ile-Pro-Pro and Val-Pro-Pro), flavanols, procyanidins, and nattokinase. These compounds are either produced or enhanced during fermentation, which improves their potency and bioavailability. For example, milk fermentation by *Lactobacillus helveticus* releases the tripeptides IPP and VPP, known for their ACE-inhibitory effects. Likewise, cocoa fermentation and microbial metabolism can increase the levels and bioaccessibility of flavanols such as epicatechin and procyanidin. In the case of soybean fermentation by *Bacillus subtilis*, nattokinase is synthesised—a fibrinolytic enzyme with established antihypertensive properties. These fermentation-induced biochemical modifications are central to the cardiovascular benefits observed in clinical trials (Table 1).

### 4.1 Bioactive peptides (IPP, VPP, 10–15 kDa and < 3 kDa MW peptides)

Several clinical trials have reported the antihypertensive effects of bioactive peptides derived from fermented dairy products, particularly isoleucine-proline-proline (IPP), valine-proline-proline (VPP), and medium and low molecular weight peptides (10–15 kDa and =3 kDa). Jauhiainen et al. (10) conducted a study involving 94 hypertensive patients who consumed 150 mL of *Lactobacillus helveticus* fermented milk daily for 4 weeks. The intervention resulted in significant reductions in both systolic (SBP) and diastolic blood pressure (DBP).

Similarly, Seppo et al. (11) reported significant SBP and DBP reductions in hypertensive volunteers (SBP ≥ 140 mmHg, DBP ≥ 90 mmHg) consuming fermented milk containing IPP and VPP peptides. In another trial, prehypertensive adults (aged 30–65 years) consumed six chewy tablets daily, each containing 1.25 g of fermented goat milk with 10–15 kDa peptides, resulting in a 12.5% reduction in SBP and a 9.3% reduction in DBP (12).

A placebo-controlled study among Japanese adults with high-normal blood pressure or mild hypertension showed that daily intake of IPP and VPP via six tablets of fermented milk led to SBP and DBP reductions of 3 and 7%, respectively, in the high-normal group, and 7 and 8% in the mild hypertension group (13). Similarly, in Japanese men aged 23–59 with borderline hypertension, the daily consumption of 160 g of fermented sour milk rich in IPP and VPP resulted in SBP and DBP reductions of 5.2 mmHg and 2.0 mmHg, respectively (14).

A study conducted in Finland among overweight or obese, middle-aged adults with mild hypertension reported a 2.6 mmHg greater SBP reduction in the group consuming sour milk containing IPP and VPP compared to the placebo group (15). Additional report comes from adults aged 39–61 with mild hypertension, where the intake of 150 g of IPP- and VPP-enriched liquid yoghurt twice daily led to progressive SBP reductions (−8.4 mmHg at week 1 and −13.9 mmHg at week 8) and DBP reductions (−6.0 mmHg at week 1 and −9.1 mmHg at week 8) (17).

In a 12-week crossover study on hypertensive adults (aged 25–55), the consumption of fermented milk containing low and high doses of tripeptides significantly reduced arterial stiffness, with an observed decrease in the augmentation index of 1.53% (18). Similarly, in adults with high-normal blood pressure (mean age: 38.3 years), the daily intake of 150 g of liquid yoghurt twice a day for 12 weeks led to notable SBP reductions (−5.5 mmHg at week 6 and −6.1 mmHg at week 12) and DBP reductions (−3.6 mmHg at week 6 and −3.8 mmHg at week 12) (19).

Fermented soy products such as miso have also demonstrated antihypertensive effects. In a study involving adults aged 40–69 years with high-normal BP or stage I hypertension, consumption of Awase miso (two servings/day) significantly reduced nighttime SBP (−9.4%) and DBP (−8.0%) compared to a soy-based control without salt, indicating the potential role of bioactive peptides < 3 kDa in miso as natural ACE inhibitors (29).

#### 4.1.1 Mechanism of action of bioactive peptides

ACE-inhibitory peptides derived from fermented milk—primarily through fermentation with *Lactobacillus helveticus*—lower blood pressure by inhibiting angiotensin-converting enzyme (ACE) activity and reducing the formation of angiotensin II (37, 38). Their mechanism of action involves binding to the active site of ACE, which is essential for the conversion of angiotensin I to angiotensin II. Studies have shown that Ile-Pro-Pro and Val-Pro-Pro possess specific structural features, particularly hydrophobic amino acids at their C-terminal ends, which enhance their affinity for ACE. This structural trait is critical for effective binding, as peptides with hydrophobic residues tend to exhibit stronger ACE-inhibitory activity (38). Moreover, these lactotripeptides demonstrate both stability and bioavailability within the digestive tract, contributing to prolonged antihypertensive effects (29, 39).

### 4.2 Polyphenols and flavanols

Several studies have highlighted the cardiovascular benefits of polyphenols and flavanols derived from fermented foods and beverages, particularly in blood pressure regulation and vascular function improvement. These compounds can be present in raw materials, but fermentation often modifies their bioavailability and bioactivity by converting large molecular weights to compounds of low molecular weight (40).

In a study involving 44 older adults, daily consumption of dark chocolate (6.3 g/day, 30 mg polyphenols) resulted in significant reductions in systolic (−2.9 mmHg) and diastolic blood pressure (−1.9 mmHg), along with improved nitric oxide (NO) bioavailability. The prevalence of hypertension declined from 86 to 68% over the study period (20).

Another study investigated the effects of dealcoholized red wine in men at high cardiovascular risk and reported significant reductions in systolic blood pressure (−5.8 mmHg) and diastolic blood pressure (−2.3 mmHg). These effects were not observed in the baseline or comparator groups and were more pronounced than those seen in participants consuming alcoholic red wine or gin, suggesting that fermentation-derived polyphenols—rather than ethanol—were responsible for the observed cardiovascular benefits (41).

In a placebo-controlled clinical trial, healthy men aged 25–32 years (BMI 19–23) consumed a high-flavanol cocoa drink containing 917 mg of total flavanols, predominantly epicatechin, and were compared to a control group receiving a low-flavanol formulation (37 mg). The intervention significantly improved vascular function, as assessed by peripheral arterial tonometry (PAT). The PAT index increased by 68.9% in the high-flavanol group compared to 18.1% in the control group (*p* = 0.004), suggesting enhanced endothelial responsiveness and nitric oxide (NO) bioactivity (16).

In healthy young adults (aged 18–24), the daily intake of 10 g of dark chocolate (>75% cocoa), rich in flavanols such as epicatechin and procyanidin, significantly improved vascular function. Outcomes included increased flow-mediated dilation (FMD 9.31%), a reduced arterial stiffness index (ASI from 0.16 to 0.13), and a reduction in pulse wave velocity (PWV from 6.13 to 5.83) (21).

#### 4.2.1 Mechanism of action of flavanols and polyphenols

Polyphenols and flavanols—such as epicatechin, catechin, procyanidin, naringenin, and hesperidin—found in dark chocolate, fermented cocoa beverages, and red wine (Table 1), contribute to cardiovascular health through multiple interconnected mechanisms. A central mode of action involves the enhancement of endothelial nitric oxide (NO) production via activation of endothelial nitric oxide synthase (eNOS), which increases NO bioavailability, promotes vasodilation, and improves vascular responsiveness (16, 42–44). This is linked to reduced arterial stiffness and a lower risk of cardiovascular disease (45).

Red wine polyphenols and dark chocolate flavanols specifically stimulate endothelial NO release, improving blood flow and reducing vascular resistance (41). In addition, these compounds exert antioxidant effects by reducing oxidative stress, inhibiting NADPH oxidase, modulating gene expression, and suppressing vasoconstrictors (41, 46).

Bioactive NO derivatives—such as S-nitrosothiols, peroxynitrite, and nitrated lipids—further support vascular function by activating soluble guanylate cyclase (sGC), increasing cyclic GMP (cGMP), and triggering vasodilation. They also display anti-inflammatory activity by neutralising cytokines like TNF-*α* and IL-6, thereby mitigating vascular inflammation and atherosclerosis risk (47, 48).

Flavanol intake has been inversely associated with cardiovascular disease risk due to its multifactorial effects: enhancing vasodilation, lowering blood pressure, improving insulin sensitivity and glucose tolerance, reducing platelet reactivity, and strengthening antioxidant defences (45, 49). These effects are partly mediated through improved endothelial function and increased NO bioactivity. Flavanols also influence redox balance, regulate gene and protein expression, and are metabolised by the gut microbiota into phenolic acids, which further contribute to their biological activity (16).

### 4.3 Nattokinase

Nattokinase is a serine fibrinolytic protease produced by *Bacillus subtilis natto* and found in fermented soy product Natto, a traditional Japanese fermented soybean product. This enzyme exhibits a positive impact on blood pressure and reduces blood clot formation (35, 50), with potential sex-specific effects. In a placebo-controlled trial involving healthy adults (aged 18–85) with elevated blood pressure, significant DBP reductions were observed in the nattokinase group compared to placebo. Men experienced a greater reduction in DBP (*p* < 0.006), while women showed a trend towards decreased waveform factor (WF, *p* < 0.1). Additionally, renin activity increased by 66% in the nattokinase group compared to 8% in the placebo group, suggesting enhanced regulation of the renin-angiotensin system (28).

#### 4.3.1 Mechanism of action of nattokinase

Nattokinase enhances plasmin activity, accelerating fibrin degradation and reducing thrombosis risk (51). Nattokinase has also been shown to lower blood pressure by inhibiting the renin-angiotensin system and modulating endothelial function (28). Additionally, this enzyme inhibits platelet aggregation, decreasing the risk of atherosclerotic plaque formation (52).

## 5 Bioactive compounds associated with lipid metabolism, cholesterol homeostasis and obesity management

Clinical studies have reported that fermented food interventions containing bioactive compounds—such as acetic acid, polyphenols (e.g., genistein, daidzein, epicatechin), isoflavones, dietary fibres, capsaicin, monacolin K, and microbial metabolites like dimerumic acid—can reduce LDL-cholesterol, total cholesterol, and triglycerides, while improving HDL-cholesterol and other metabolic markers. Fermentation enhances the concentration, transformation, or bioavailability of these key compounds. For example, *Pichia kluyveri* fermentation of orange juice increases flavonoid content; red yeast rice fermentation produces monacolin K, a compound with lipid-lowering properties; fermented soy products concentrate isoflavones and generate anti-obesogenic peptides; and fermentation of oats and tempeh improves fibre solubility, supporting cholesterol metabolism through increased production of short-chain fatty acids (SCFAs). These fermentation-mediated transformations underpin the lipid-regulating effects observed in clinical trials.

### 5.1 Acetic acid

A double-blind placebo-controlled trial in hypercholesterolaemic adults (ages 35–60) assessed the effects of daily supplementation with 30 mL of red date vinegar over 8 weeks. The intervention resulted in significant reductions in total cholesterol (−19.8%; 197.6 g), LDL-C (−34.9%; 109.8 g), and TG (−5.3%; 161.2 mg/dL), alongside a 5.75% increase in HDL-C (44.1 g) (53).

#### 5.1.1 Mechanism of action of acetic acid

Acetic acid acts as a key metabolic substrate, converted to acetyl-CoA in hepatocytes, where it activates AMP-activated protein kinase (AMPK)—a central regulator of energy metabolism. This activation suppresses fatty acid synthesis, lowers malonyl-CoA concentrations, and promotes *β*-oxidation (54). Furthermore, acetic acid enhances bile acid excretion and inhibits sterol regulatory element-binding proteins (SREBPs), collectively supporting lipid-lowering effects (55).

### 5.2 Polyphenols and flavonoids

In a clinical trial involving nine healthy adults, the effects of fermented orange juice (FOJ) were compared to regular orange juice (OJ). Following a two-week washout period, FOJ consumption resulted in more favourable lipid responses, including a smaller increase in LDL-C (621.9 vs. 718.6 mg/dL) and a significant reduction in apolipoprotein B (apo-B: −409.7 ± 23.1), supporting the hypolipidemic potential of FOJ-derived flavonoids (56). In a separate crossover study involving 30 healthy adults, daily consumption of 500 mL of fermented orange juice rich in flavonoids and vitamin C over several weeks led to significant improvements in oxidative and inflammatory biomarkers. Antioxidant capacity, measured by ORAC, increased by 43.9%, uric acid levels decreased by 8.9%, and lipid peroxidation markers (TBARS) were reduced by 30.2% (57).

In another study, adult males (aged 45–50) who consumed 272 mL/day of dealcoholised red wine showed a significant increase in urinary phenolic metabolites (*p* < 0.05), suggesting enhanced bioavailability and systemic engagement with lipid metabolic pathways (58). Similarly, in a two-week trial involving 73 healthy adults (aged 20–50), daily intake of 25 g of dark chocolate twice a day led to a 6% increase in HDL-C (p < 0.05), underscoring the lipoprotein-modulating potential of cocoa-derived flavanols (22).

#### 5.2.1 Mechanism of action of polyphenols and flavonoids

Polyphenols regulate lipid metabolism through several complementary mechanisms. Flavonoids such as epicatechin activate AMP-activated protein kinase (AMPK), thereby promoting fatty acid oxidation and suppressing hepatic triglyceride synthesis (59). Additionally, the microbial fermentation of polyphenols and co-ingested dietary fibres in the gut yields short-chain fatty acids (SCFAs), which may enhance HDL-C functionality and facilitate cholesterol efflux through the upregulation of specific transporters (60, 61). The antioxidant and anti-inflammatory properties of polyphenols further contribute to improved endothelial function and metabolic homeostasis.

Specifically, in fermented orange juice, the 10-day alcoholic fermentation process using *Pichia kluyveri* improves the bioavailability and metabolic effects of flavonoids and phenolic compounds (56). These compounds also stimulate endogenous antioxidant responses (e.g., uric acid, bilirubin), inhibit *α*-glucosidase activity to support glucose regulation, and improve lipid profiles by reducing LDL-C and apo-B levels (56, 62, 63).

### 5.3 Isoflavones and small peptides

A placebo-controlled study in overweight or obese adults (ages 19–65) investigated the effects of a daily 9.8 g dose of freeze-dried doenjang—a fermented soy food containing genistein, daidzein, and small peptides. Participants carrying the PPAR-*γ* C allele demonstrated the most pronounced response, with catalase (CAT) antioxidant enzyme activity doubling post-intervention (30).

#### 5.3.1 Mechanism of action of isoflavones and peptides

Isoflavones such as genistein and daidzein activate peroxisome proliferator-activated receptors (PPAR-*α* and PPAR-γ), modulating lipid oxidation, glucose uptake, and adipocyte differentiation (64). In parallel, small peptides derived from fermentation inhibit adipogenesis and improve lipid distribution by suppressing transcription factors like SREBP-1c and fatty acid synthase (FAS), reducing body fat accumulation (65).

### 5.4 Soluble and insoluble dietary fibre

In a study of 41 hyperlipidaemic women, daily consumption of tempeh gembus at two doses (103 g and 206 g) for several weeks led to reductions in LDL-C (−27.9, −30.9%) and total cholesterol (−17.7, −19.8%), with corresponding increases in HDL-C (+3.91%, +8.79%) and minor increases in TG (+2.3%, +3.1%) (31).

Swedish adults (ages 23–71) who consumed 600 mL/day of *β*-glucan-rich fermented oat beverages experienced a 6% reduction in total cholesterol (66).

#### 5.4.1 Mechanism of action of dietary fibre

Soluble fibres such as β-glucan bind bile acids in the intestine, promoting their excretion and triggering hepatic conversion of cholesterol into new bile acids. Fermentation of fibres by gut microbiota produces SCFAs—particularly propionate and butyrate—which inhibit hepatic cholesterol synthesis and modulate systemic lipid metabolism (2, 67).

### 5.5 Lactoferrin, beta-lactoglobulin and exopolysaccharides

In a randomised, placebo-controlled trial involving 161 hypercholesterolaemic adults (mean age 54.5 ± 9.8 years), daily intake of a malleable protein matrix (MPM) derived from fermented whey proteins resulted in a 9.8% reduction in serum triglycerides compared to a gelatin-based placebo group (68).

#### 5.5.1 Mechanism of action of fermented whey matrix

MPM contains lactoferrin, beta-lactoglobulin, and exopolysaccharides, which modulate lipid metabolism by inhibiting intestinal fat absorption and enhancing faecal sterol excretion (69). Additionally, branched-chain amino acids in whey regulate hepatic gene expression related to lipid synthesis and cholesterol metabolism.

### 5.6 Capsaicin, genistein, and daidzein

In one study with overweight or obese adults (aged 19–65), 32 g/day of kochujang paste significantly reduced TG (−14.1%) and TG/HDL ratio (−13.6%), with improvements in insulin sensitivity (*p* < 0.05) (32). Another trial in healthy adults (aged 19–55) reported that 34.5 g/day of kochujang pills led to a 9.75% reduction in total cholesterol compared to placebo (33).

#### 5.6.1 Mechanism of action of capsaicin and isoflavones

Capsaicin activates transient receptor potential vanilloid 1 (TRPV1) channels, triggering AMPK activation and enhancing fatty acid oxidation. Isoflavones inhibit SREBP-1c and FAS, reducing lipogenesis and cholesterol synthesis via downregulation of HMG-CoA reductase activity (70, 71).

### 5.7 Monacolin K and dimerumic acid

A clinical study in adults (aged 26–68) with borderline hyperlipidaemia (TG: 120–200 mg/dL) demonstrated that supplementation with *Monascus pilosus*-fermented garlic extract (MGFE) significantly reduced TG (−14.9%), total cholesterol (−8.6%), and LDL-C (−14.2%) compared to placebo (72).

#### 5.7.1 Mechanism of action of monacolin K and dimerumic acid

Monacolin K mimics the activity of statins by competitively inhibiting HMG-CoA reductase, a key enzyme in endogenous cholesterol synthesis. Dimerumic acid contributes additional lipid-lowering effects by acting as a potent antioxidant, thereby reducing lipid peroxidation and improving lipid profile stability (72, 73).

### 5.8 Phenolic compounds

A clinical study involving normal-weight and obese adults (aged 18–45) reported that daily consumption of 200 mL of kombucha—rich in phenolic compounds such as theaflavins, thearubigins, quercetin, catechin, kaempferol, and their derivatives—led to a significant increase in *Subdoligranulum*, a gut microbial genus potentially associated with improved lipid metabolism and glycaemic regulation (*p* = 0.031) (74).

#### 5.8.1 Mechanism of action of phenolic compounds

Phenolic compounds of kombucha are largely unabsorbed in the small intestine and reach the colon intact, where they act as prebiotics. They enhance microbial diversity and SCFA production, particularly butyrate and acetate, which modulate lipid absorption, maintain gut barrier integrity, and suppress inflammatory responses (74).

## 6 Bioactive compounds associated with antioxidant activity and oxidative stress reduction

Fermented foods provide bioactive compounds—such as polyphenols (e.g., epicatechin, procyanidins) and GABA—that have demonstrated systemic antioxidant effects in clinical trials. These effects include increased plasma antioxidant capacity, reduced lipid peroxidation, and enhanced enzymatic antioxidant defences. Fermentation improves the solubility, stability, and physiological activity of these compounds. For instance, cocoa fermentation increases the content of extractable flavanols while reducing pro-oxidant constituents. Fermented papaya preparation (FPP) generates low-molecular-weight antioxidants with synergistic redox activity. Additionally, *Lactobacillus brevis* fermentation of sea tangle enhances GABA bioavailability, amplifying antioxidative responses. These fermentation-driven improvements contribute to the observed enhancement of redox status in human participants.

### 6.1 Polyphenols (procyanidin and epicatechin)

Multiple studies have shown that fermented cocoa-based products, particularly chocolate and cocoa beverages, significantly enhance plasma antioxidant status and reduce oxidative damage. In a dose–response trial conducted in healthy adults (aged 20–56 years), intake of procyanidin-rich chocolate (27, 53, and 80 g) led to a dose-dependent increase in plasma epicatechin levels by 133, 258, and 355%, respectively. Lipid peroxidation (TBARS) decreased by 20% at the highest dose, and total antioxidant capacity was significantly improved, with an average increase of 253 s in the antioxidant capacity assay (23).

In a separate study, healthy adults (aged 18–50 years, BMI 21.6) consumed a cocoa beverage (40 g cacao powder) following a polyphenol-free washout. The intervention significantly enhanced total antioxidant activity by 62.35% (*p* < 0.0073) compared to the control (24).

Additionally, fermented fruit-based formulations have demonstrated systemic antioxidant benefits. In a clinical trial with neo-diabetic adults (aged 25–60), daily supplementation with fermented papaya preparation (FPP®) reduced high-sensitivity C-reactive protein (CRP) by 13.3%, improved the LDL/HDL ratio by 2.6%, and significantly lowered uric acid levels (*p* < 0.001), indicating a favourable shift in redox and inflammatory status (75).

#### 6.1.1 Mechanism of action of polyphenols

Epicatechin, a flavanol abundant in cocoa, acts via direct and indirect antioxidant mechanisms. It scavenges reactive oxygen species (ROS), inhibits lipid peroxidation, and enhances total antioxidant capacity (23). It also upregulates endogenous antioxidant enzymes and reduces oxidative stress by inhibiting pro-oxidant enzymes and modulating phase II detoxification enzymes (24, 76).

Fermented papaya preparation exerts antioxidant effects through the synergistic action of polyphenols and sulphur-containing amino acids, enhancing glutathione synthesis and modulating oxidative stress markers such as CRP and uric acid (77). These effects support vascular integrity and metabolic health in oxidative stress-prone individuals.

### 6.2 GABA

GABA-enriched fermented foods have also been associated with systemic antioxidant enhancement. In a placebo-controlled trial involving healthy adult males (aged 25–60 years) with elevated *γ*-glutamyl transferase (γ-GT), participants consumed fermented sea tangle (FST) daily. Significant reductions were observed in serum γ-GT (−23.51 U/L) and malondialdehyde (MDA; −10.47 qmol/mg), indicating lowered oxidative burden. Concurrently, enzymatic antioxidant defences improved: superoxide dismutase (SOD) increased by 49.60 U/mL and catalase (CAT) by 13.64 mU/mL (78).

#### 6.2.1 Mechanism of action of GABA

GABA exerts antioxidant effects via multiple routes, including enhancing enzymatic antioxidant defence (SOD, CAT, and glutathione peroxidase) and reducing oxidative biomarkers like MDA. It modulates cellular redox status and promotes homeostasis by inhibiting oxidative stress pathways (79). Furthermore, fermentation with *Lactobacillus brevis* increases GABA yield and bioavailability, potentiating its antioxidative and hepatoprotective functions (78).

## 7 Bioactive compounds associated with glucose metabolism and insulin sensitivity

Bioactive compounds in fermented foods—including organic acids, dietary fibres, polyphenols, methylxanthines, *γ*-polyglutamic acid (γ-PGA), and phenolic acids—have demonstrated significant effects on glycaemic regulation and insulin sensitivity in clinical trials. Fermentation modulates the physiological activity, structure, and availability of these compounds. For example, sourdough fermentation increases lactic and acetic acid levels, enhancing insulin receptor sensitivity; cocoa fermentation elevates the concentration of bioavailable epicatechin; natto fermentation generates high-viscosity γ-PGA, which delays glucose absorption; and rye fermentation promotes the release of free phenolic acids that improve insulin signalling pathways. These fermentation-induced biochemical modifications collectively enhance the glycaemic control efficacy of fermented food interventions.

### 7.1 Organic acids and dietary fibre

In a randomised crossover trial involving 16 healthy adults (6 males and 10 females; aged 20–50), sourdough bread rich in insoluble dietary fibre and fermentation-derived lactic and acetic acids was compared with commercial par-baked wheat bread. The sourdough intervention significantly reduced postprandial glucose area under the curve (AUC) by 8% and insulin AUC by approximately 26%, demonstrating enhanced insulin sensitivity and improved glycaemic regulation (80).

#### 7.1.1 Mechanism of action of organic acids and dietary fibre

Insoluble dietary fibre exerts glycaemic control by physically entrapping glucose and digestive enzymes (e.g., *α*-amylase, α-glucosidase), reducing glucose availability and delaying carbohydrate digestion (81, 82). Additionally, organic acids such as lactic and acetic acids modulate interstitial fluid pH, improving insulin receptor activity and preventing insulin resistance in muscle and adipose tissue (83, 84).

### 7.2 Epicatechin and theobromine

A crossover study with 19–38-year-old male cyclists evaluated the acute metabolic impact of a high-cocoa dark chocolate supplement (561 kcal; 89 mg epicatechin, 690 mg theobromine) compared to a cocoa-depleted control. The intervention led to an 8% increase in plasma glucose and a 15% increase in muscle glycogen utilisation, accompanied by an 18% reduction in glucose oxidation. These findings indicate enhanced glucose availability and muscular uptake, supporting metabolic efficiency during physical exertion (25).

#### 7.2.1 Mechanism of action of epicatechin and theobromine

Epicatechin modulates glucose homeostasis by stimulating nitric oxide (NO) production and enhancing GLUT4 translocation in skeletal muscle, thereby facilitating glucose uptake. Theobromine, a methylxanthine, complements this action by regulating phosphodiesterase activity and cyclic AMP levels, enhancing insulin signalling and glucose metabolism (25).

### 7.3 Caffeic, ferulic, and sinapic acids

In a randomised trial involving 21–28-year-old healthy adults, the consumption of rye breads, rich in phenolic acids (e.g., caffeic, ferulic, sinapic), resulted in significantly lower glycaemic indices compared to white wheat bread (WWB). These fermented rye varieties also demonstrated insulin-sparing properties, suggesting improved glucose handling and postprandial regulation (85).

#### 7.3.1 Mechanism of action of caffeic, ferulic, and sinapic acids

Phenolic acids such as caffeic, ferulic, and sinapic acids exert glycaemic control through multiple biochemical pathways. These hydroxycinnamic acids inhibit carbohydrate-digesting enzymes, particularly *α*-amylase and α-glucosidase, thereby delaying starch hydrolysis and reducing the postprandial glucose surge, ultimately contributing to lower glycaemic indices (86, 87). Additionally, ferulic and caffeic acids activate AMP-activated protein kinase (AMPK) in peripheral tissues, enhancing glucose uptake and promoting GLUT4 translocation in skeletal muscle, which improves insulin sensitivity (88, 89). These compounds also possess antioxidant properties that mitigate oxidative stress-induced insulin resistance by scavenging reactive oxygen species (ROS), reducing lipid peroxidation, and modulating inflammatory cytokines such as TNF-α and IL-6 (90). Their combined effects on enzymatic inhibition, cellular glucose transport, and redox balance support improved postprandial glycaemic regulation and explain the insulin-sparing outcomes observed in fermented rye bread interventions.

### 7.4 Polyglutamic acid (*γ*-PGA)

In a controlled trial with men and postmenopausal women, participants consumed high-γ-PGA natto (439.6 mg γ-PGA/40 g) or a low-γ-PGA variant (57.6 mg γ-PGA/40 g). The high-γ-PGA group experienced significant reductions in glucose incremental AUC at 0–15 and 0–30 min (*p* < 0.001), alongside a decrease in insulin levels at 0–45 min (*p* < 0.01), indicating improved glycaemic control and insulin economy (34).

#### 7.4.1 Mechanism of action of polyglutamic acid

γ-PGA, a fermentation-derived viscous biopolymer, slows glucose absorption by increasing food viscosity and forming a gel-like matrix in the gastrointestinal tract. This physical barrier effect attenuates postprandial glycaemic spikes and may mimic the function of soluble fibres such as *β*-glucans. High-viscosity γ-PGA has also been linked to reduced dysglycaemia risk and improved early-phase glucose regulation (34, 91).

## 8 Bioactive compounds affecting haematological parameters

Fermented food bioactive compounds—epicatechin, flavan-3-ols, whey protein-derived peptides, and nattokinase—show beneficial effects on haematological markers in clinical studies, including improved red blood cell indices, platelet aggregation inhibition, and enhanced fibrinolysis. Fermentation enhances their potency and availability: cocoa fermentation improves flavanol bioaccessibility, contributing to antiplatelet effects; whey protein fermentation increases peptide release, supporting erythropoiesis; and *Bacillus subtilis* soybean fermentation produces nattokinase, a fibrinolytic enzyme absents in unfermented soybeans. These transformations enhance the compounds’ bioefficacy for haematological health.

### 8.1 Epicatechin

Epicatechin, a flavanol, has been shown to influence platelet function. In a study involving 18 healthy males, the acute consumption of 50 g of high-flavanol dark chocolate resulted in a positive correlation between plasma flavanol metabolites and reduced platelet aggregation, indicating a potential for reduced thrombotic risk (26).

#### 8.1.1 Mechanism of action of epicatechin

Epicatechin improves vascular and haematologic function by enhancing nitric oxide (NO) bioavailability through endothelial nitric oxide synthase (eNOS) activation, which supports vasodilation and reduces arterial stiffness (92). In haematological pathways, epicatechin-sulfate metabolites suppress platelet glycoprotein IIb/IIIa activation, thereby reducing aggregation (93).

### 8.2 Flavan-3-ols and catechins

A randomised crossover study assessed the effects of dark chocolate formulations enriched with flavan-3-ols and catechins in healthy adults aged 23–65. Participants consumed 60 g of dark chocolate per treatment, and significant improvements in platelet reactivity were observed. Dark chocolate improved platelet function in both men (*p* ≤ 0.020) and women (*p* ≤ 0.041) with an improved platelet function in men (*p* = 0.002), suggesting sex-dependent differences in response (27).

#### 8.2.1 Mechanism of action of flavan-3-ols and catechins

Flavan-3-ols and catechins modulate haemostasis by inhibiting ADP- and collagen-induced platelet adhesion and aggregation (26). Their antioxidant activity increases NO bioavailability by inhibiting NADPH oxidase and vasoconstrictor endothelin-1. NO in turn downregulates NOX2, reducing platelet isoprostane generation and fibrinogen binding to GpIIb/IIIa, thereby improving vascular health and reducing clotting risk (94, 95).

### 8.3 Bioactive peptides

Whey protein-derived bioactive peptides, delivered via high-protein fermented yoghurt, have demonstrated haematologic benefits in athletes. In a 9-week study, 24 young athletes consumed yoghurt enriched with either 10% or 20% whey protein isolate (WPI) three times daily. Results showed a significant increase in haemoglobin levels: +1.15 g/dL in the 10% group and +1.8 g/dL in the 20% group, supporting the erythropoietic effects of peptides in functional dairy matrices (42).

#### 8.3.1 Mechanism of action of bioactive peptides

Whey-derived peptides stimulate muscle protein synthesis and haemoglobin production due to their high branched-chain amino acid (BCAA) content, particularly leucine (96). Fermentation enhances their bioactivity by increasing antioxidant peptide production (97). These peptides promote myogenesis and protect against oxidative muscle damage, while improving protein synthesis and reducing degradation (98, 99).

### 8.4 Nattokinase

Nattokinase demonstrated strong anticoagulant and fibrinolytic activity. In a single-dose clinical trial with healthy males (mean age: 22.3 years), nattokinase (2000 FU) significantly increased D-dimer levels by 44.5% (at 6 h) and fibrin degradation products (FDP) by 21.2% (at 4 h). It also prolonged activated partial thromboplastin time (aPTT) significantly (*p* < 0.05), indicating reduced coagulation and enhanced fibrinolysis (35).

#### 8.4.1 Mechanism of action of nattokinase

Nattokinase accelerates fibrin clot degradation by directly cleaving cross-linked fibrin and enhancing tissue plasminogen activator (tPA) activity while inhibiting plasminogen activator inhibitor-1 (PAI-1) (51). It also reduces thrombin and Factor Xa activity, increases antithrombin levels, and lowers Factor VIII, offering both anticoagulant and fibrinolytic effects with prolonged activity (35, 52).

## 9 Bioactive compounds associated with immune modulation and inflammation reduction

Clinical studies demonstrate that fermented food bioactive compounds, primarily conjugated linoleic acid (CLA) and arabinoxylan, modulate immune function and reduce inflammation. Fermentation enhances their presence and functionality: cheese ripening enriches CLA through microbial conversion of linoleic acid, while rice bran fermentation increases arabinoxylan bioavailability. These modifications enable enhanced cytokine response regulation, immune cell activation, and anti-inflammatory signalling, contributing to reduced TNF-*α*, IL-6, IL-8, and elevated IFN-*γ* in human trials.

### 9.1 CLA

In a randomised controlled trial conducted among healthy Italian adults (aged 30–65 years), weekly consumption of 200 g of CLA containing Pecorino Toscano cheese led to significant reductions in inflammatory cytokines. Compared to the placebo group consuming an equivalent portion of commercial cheese, the intervention group experienced a 43% reduction in interleukin-6 (IL-6), a 36% decrease in interleukin-8 (IL-8), and a 36% reduction in tumour necrosis factor-alpha (TNF-α) levels (100). These outcomes support the anti-inflammatory role of CLA, suggesting that regular intake of CLA-containing fermented dairy can modulate lipid signalling pathways and cytokine gene expression, thereby attenuating chronic low-grade inflammation.

#### 9.1.1 Mechanism of action of CLA

Polyunsaturated fatty acids (PUFAs), including CLA, serve as precursors for inflammatory eicosanoids and are involved in regulating immune cell activity. CLA modulates immune responses by altering intracellular signalling and reducing immunoglobulin E (IgE) levels, thereby exerting anti-allergic and anti-inflammatory effects (101–103). Furthermore, CLA influences cytokine production and immune cell differentiation by interacting with nuclear receptors and downstream transcription factors, which regulate inflammation-related gene expression (104).

### 9.2 Arabinoxylan

In another double-blind, placebo-controlled study, 80 healthy individuals (aged 25–70 years) were supplemented with arabinoxylan-rich extract derived from fermented rice bran (RBEP) for 8 weeks. Participants in the intervention group exhibited significantly elevated levels of interferon-gamma (IFN-*γ*) (35.56 ± 17.66 pg./mL) compared to the placebo group (27.04 ± 12.51 pg./mL; *p* = 0.012), suggesting enhanced immune activation (105).

#### 9.2.1 Mechanism of action of arabinoxylan

Arabinoxylans act as potent immunomodulatory agents by stimulating natural killer (NK) cell activity and enhancing the expression of activation markers CD25 and CD69. These oligosaccharides also promote the secretion of pro-inflammatory cytokines such as tumour necrosis factor-alpha (TNF-*α*) and IFN-γ through the upregulation of interleukin-2 (IL-2), facilitating immune cell expansion and cytotoxic function (106–108). Moreover, their prebiotic properties foster the production of short-chain fatty acids (SCFAs) by gut microbiota, which contribute to immune system regulation and the reinforcement of mucosal barriers (109, 110).

## 10 Bioactive compounds with neuroprotective and cognitive health effects

Emerging evidence indicates fermented food bioactive compounds can positively influence neurocognitive functions. The most studied, GABA, increases significantly during fermentation—especially in beverages and plant-based substrates—through *Lactobacillus brevis* metabolic activity. This microbial conversion enhances GABA concentration and bioavailability. Clinically, GABA-enriched interventions improve anxiety, sleep latency, and stress-related biomarkers, highlighting fermentation-enhanced GABA’s neuromodulator potential.

### 10.1 GABA

In a placebo-controlled study involving 17 healthy females (mean age 40.9 ± 2.56), daily intake of fermented beverages containing GABA led to notable improvements in sleep quality and stress-related parameters. Participants reported a 40% reduction in sleep latency, a 27% reduction in total physical activity during sleep, and a 12.5% decrease in state anxiety scores (all *p* ≤ 0.05), indicating GABA’s role in promoting sleep and mental calmness (111).

#### 10.1.1 Mechanism of action of GABA

GABA is the primary inhibitory neurotransmitter in the central nervous system and plays a critical role in reducing neuronal excitability and promoting neural homeostasis. It functions by binding to GABA_A and GABA_B receptors. GABA_A receptors facilitate chloride ion (Cl^−^) influx, resulting in neuronal hyperpolarisation and suppression of nerve firing, thereby producing anxiolytic and calming effects. GABA_B receptors activate G-protein-coupled pathways, promoting K^+^ efflux and Ca^2+^ channel inhibition, further reducing synaptic transmission and excitability (112, 113).

Additionally, GABA contributes to antioxidant defences by lowering lipid peroxidation (e.g., reduced MDA), increasing superoxide dismutase (SOD) and glutathione peroxidase (GPx) activities, and preventing advanced lipoxidation end-product formation (79). These effects protect critical brain regions such as the hippocampus and cerebral cortex, which are essential for memory and mood regulation (114, 115).

## 11 Bioactive compounds associated with liver health and detoxification

Fermented food bioactive compounds—primarily curcumin and ginsenosides (compound K)—demonstrate hepatoprotective effects by modulating liver enzymes, reducing inflammation, and enhancing antioxidant defences. Both compounds undergo biochemical transformation during fermentation: *Aspergillus oryzae* turmeric fermentation converts curcumin to lower molecular weight compounds with improved solubility and absorption; ginseng fermentation converts native ginsenosides to compound K with enhanced intestinal uptake. These fermentation-enhanced transformations contribute significantly to observed clinical benefits on liver health and detoxification.

### 11.1 Curcumin

In a 12-week double-blind randomised controlled trial (DB-RCT), adults aged 20–70 years with elevated alanine aminotransferase (ALT >40 IU/L) consumed 3.0 g/day of fermented turmeric powder (FTP) produced using *Aspergillus oryzae*. The intervention significantly reduced serum liver enzymes: ALT decreased by 26.5% and aspartate aminotransferase (AST) by 23.1% from baseline values (116). Improvements were also observed in lipid peroxidation biomarkers and liver histopathology.

#### 11.1.1 Mechanism of action of curcumin

Fermentation enhances curcumin’s solubility and bioavailability, facilitating its hepatoprotective action. Curcumin activates the nuclear factor erythroid 2-related factor 2 (Nrf2) pathway, promoting the expression of endogenous antioxidant enzymes such as glutathione (GSH), glutathione peroxidase (GPx), and superoxide dismutase (SOD), thereby reducing oxidative stress (117). Additionally, it downregulates nuclear factor kappa B (NF-κB) and signal transducer and activator of transcription (STAT) proteins, attenuating inflammatory cytokines and preventing hepatic injury (77). These actions collectively contribute to liver enzyme regulation and systemic detoxification.

### 11.2 Ginsenosides (compound K)

A separate 12-week DB-RCT examined the effects of fermented ginseng extract containing ginsenoside compound K in adults with elevated ALT (aged 19–70 years). Participants receiving either 125 mg/day or 500 mg/day of GBCK25 showed a 13.5 IU/L reduction in gamma-glutamyl transferase (GGT; *p* = 0.036) and a significant decrease in high-sensitivity C-reactive protein (hs-CRP; −1.51 mg/L, *p* = 0.021), compared to placebo (118). Fatigue scores also improved, suggesting both physiological and subjective benefits.

#### 11.2.1 Mechanism of action of compound K

Compound K, a metabolite formed during ginseng fermentation, exerts anti-inflammatory and hepatoprotective effects through its steroid-like structure that enables binding to glucocorticoid receptors (GR). This interaction downregulates NF-κB transcription, reducing pro-inflammatory cytokines such as IL-6, IL-8, and IL-1β, as well as markers like hs-CRP and GGT (119). By modulating inflammatory and oxidative stress pathways, fermented GCK supports hepatic homeostasis and systemic detoxification (120).

## 12 Bioactive compounds associated with other health benefits

Clinical studies report diverse effects of fermented foods beyond major health domains, including improved gut function, kidney stone prevention, muscle preservation, visual performance, and anticancer activity. The responsible bioactive compounds—prebiotic oligosaccharides, glycosylceramides, acetic acid, enterolignans, isoflavones, and anthocyanins—are uniquely generated or enhanced during fermentation. *Aspergillus oryzae* fermentation enzymatically produces oligosaccharides and glycosylceramides improving gut barrier function; grain and soy fermentation boosts isoflavones and enterolignans; vinegar fermentation produces acetic acid modulating renal function. These transformations create functional interventions targeting specific health outcomes beyond primary metabolic domains.

### 12.1 Prebiotic oligosaccharides and glycosylceramides

In a randomised, double-blind clinical trial involving 44 healthy adults (18 males, 26 females), daily intake of 118 g of koji amazake—a fermented rice beverage produced with *Aspergillus oryzae*—led to statistically significant improvements in bowel function. Defecation frequency increased from 4.18 to 5.41 times per week, and faecal weight rose from 501 g to 724 g over a 3-week period. The intervention also modulated gut microbiota composition, with a decrease in *Blautia* and an increase in *Bacteroides* abundance, indicating favourable shifts in microbial balance and fermentation activity (121).

#### 12.1.1 Mechanism of action of prebiotic oligosaccharides and glycosylceramides

The oligosaccharides, including panose, kojibiose, and isomaltose—prebiotic sugars generated by *A. oryzae* enzymatic activity and glycosylceramides (GlcCer) containing a fungal-specific C9-methylated sphingoid base in koji amazake act as prebiotic substrates for colonic microbiota, promoting SCFA production and altering microbial composition (121). Produced SCFAs—particularly acetate and butyrate—support gut barrier integrity, energy metabolism in colonocytes, and systemic immune modulation, and glycosylceramides (GlcCer) from *A. oryzae* (*Koji amazake* contained 1.39 mg of GlcCer, including 1.16 mg from *A. oryzae*) may further support intestinal epithelial function and lipid metabolism (121). These compounds play key roles in maintaining gut health by supporting colonocyte energy metabolism, promoting mucin and tight junction protein expression, and reducing intestinal permeability. SCFAs also modulate immune responses through G-protein-coupled receptors and enhance mineral absorption, gut-brain communication, and metabolic regulation (122, 123).

### 12.2 Acetic acid

In a three-month, randomised clinical trial involving 72 individuals with a history of calcium oxalate (CaOx) kidney stones, participants consumed 5 mL of acetic acid-based fermented vinegar three times daily. The study assessed the impact of vinegar consumption on nephrolithiasis recurrence over a 12-month period using longitudinal monitoring. Renal ultrasound examinations were conducted at 3, 6, and 12 months, and non-contrast computed tomography (CT) was performed if renal calculi were detected during screening. Urine and serum samples were collected at baseline and follow-up to evaluate biochemical changes. The intervention group exhibited a reduced recurrence of CaOx kidney stones compared to controls, indicating a protective effect of fermented vinegar on nephrolithiasis risk (124).

#### 12.2.1 Mechanism of action of acetic acid

Acetic acid modulates renal stone risk by altering urinary pH and promoting citrate excretion, a key inhibitor of calcium oxalate (CaOx) crystal aggregation. Enhanced citrate levels chelate free calcium ions, thereby reducing urinary supersaturation of oxalate and inhibiting crystal nucleation and growth. Additionally, acetic acid influences renal epithelial physiology through epigenetic mechanisms, including increased histone H3 acetylation and upregulation of specific microRNAs that downregulate calcium transport-related genes. These epigenetic modifications lead to reduced renal calcium handling and a lower propensity for stone formation. Additionally, acetic acid supports gut microbial diversity and strengthens renal epithelial barrier integrity by enhancing occludin expression (124, 125).

### 12.3 Enterolignans (enterolactone, enterodiol, matairesinol)

A randomised clinical trial conducted on prostate cancer patients investigated the effect of consuming 155 g/day of fermented rye bran bread. Compared to control (135 g/day), the intervention/exposure group showed a 46.42% increase in apoptotic activity (TUNEL assay), a 14.58% increase in proliferation index (Ki-67), and a 3.42% reduction in the cell cycle inhibitor p27, suggesting anti-cancer activity (126).

#### 12.3.1 Mechanism of action of enterolignans

Enterolignans act as phytoestrogens, modulating oestrogen receptor pathways and gene expression involved in apoptosis, cell proliferation, and hormonal balance (127). These effects may be relevant in hormone-sensitive cancers such as prostate cancer.

### 12.4 Isoflavones

In a large-scale population-based study involving 351 individuals with type 2 diabetes (192 men and 159 women), regular consumption of fermented soy-based foods rich in isoflavones was associated with a significantly reduced prevalence of sarcopenia. Among miso consumers, only 18.8% were sarcopenic, compared to 42.3% in non-consumers, indicating a protective effect against muscle loss (36).

#### 12.4.1 Mechanism of action of isoflavones

Isoflavones such as genistein and daidzein mimic oestrogenic activity, which supports muscle protein synthesis and reduces inflammatory cytokines like TNF-*α* and IL-6. They also regulate adipose tissue metabolism, reduce visceral fat, and enhance insulin sensitivity—factors that collectively protect against sarcopenia and metabolic deterioration (36, 128).

### 12.5 Anthocyanins

In a four-week clinical trial involving myopic adults aged 31–53, daily supplementation with 400 mg of fermented bilberry extract led to improvements in visual performance. The amplitude of accommodation increased from 4.62 ± 1.88 to 5.33 ± 2.03 diopters (D), while the area under the log contrast sensitivity function (AULCSF) improved from 1.04 ± 0.16 to 1.13 ± 0.17, suggesting better visual acuity and low-light contrast detection (129).

#### 12.5.1 Mechanism of action of anthocyanins

Anthocyanins enhance vision through multiple mechanisms, including acceleration of rhodopsin resynthesis and modulation of retinal enzymatic activity. They also improve retinal microcirculation, ensuring adequate nutrient and oxygen delivery to the retina, which supports visual processing and photoreceptor responsiveness in low-light environments (129, 130).

## 13 General discussion

This systematic review catalogued bioactive compounds reported in 50 scientific papers based on human clinical studies that evaluated the health effects of compounds that were either generated, increased in quantity, or transformed into more bioavailable forms—even within the same class of compounds in raw matrix—through fermentation. Studies involving complex bioactive profiles, such as phenolic compounds, polyphenols, isoflavones, and their metabolites, were included if specific compounds dominant within the overall composition were identified, or if the health effects could reasonably be attributed to fermentation-induced compositional changes.

The analysis of these studies revealed recurring patterns across bioactive compound classes, intervention designs, and health outcome categories. Peptides and polyphenols emerged as the most frequently and consistently studied bioactive compound classes. Peptides, particularly bioactive tripeptides like IPP and VPP derived from lactic acid fermentation of milk, demonstrated robust antihypertensive effects across multiple trials (10, 11, 13, 15, 17). Similarly, polyphenols—including flavanols, anthocyanins, and flavonoids—exhibited broad-spectrum benefits ranging from cardiovascular protection (20, 21, 41) to lipid modulation (53, 58), antioxidant defence (24, 57, 75), neuroprotection (111), and visual function enhancement (129). Fermentation was found to enhance the stability and intestinal absorption of polyphenols, particularly through microbial deglycosylation and metabolite transformation. Dietary fibres, especially *β*-glucans and insoluble fibres from oats, rye, and tempeh (31, 66, 80), found in fermented foods, played a prominent role in glycaemic regulation, cholesterol reduction, and gut health promotion.

In terms of fermentation types, lactic acid fermentation was the most dominant, employed in the production of fermented milk, yoghurt, kimchi, miso, and fermented sea tangle. This fermentation modality is notable not only for its safety and widespread application in food cultures but also for its capacity to generate short-chain peptides and GABA with well-documented bioactivities. Alcoholic and acetic acid fermentations were also represented involving dealcoholized red wine (41), kombucha (74), red date vinegar (53), and fermented orange juice (56, 57) (Supplementary Table 1). These methods were associated with increased polyphenol bioavailability, antioxidant effects, and metabolic modulation.

Across the included studies, dose standardisation and enhancement of bioavailability via fermentation were recurrent features, although not uniformly implemented. Several trials administered defined dosages of peptides (e.g., IPP/VPP in mg per 100 mL of milk), polyphenols (e.g., quantified anthocyanin content in fermented bilberry extract), or GABA (e.g., 1.5 g/day in fermented sea tangle). However, not all studies provided comprehensive dose–response analyses. Fermentation was frequently used as a tool to enhance the functionality and uptake of these compounds—examples include the use of LAB strains to increase *γ*-PGA viscosity in natto (34), or microbial enzymatic transformation of ginsenosides into compound K in fermented ginseng (118).

Overall, the findings suggest that the bioactive compounds presented in this review—whether derived, enhanced, or modulated under different fermentation conditions and present in the final fermented food products—may exert significant health effects across various health domains when used as part of an intervention or exposure in different populations.

## 14 Limitations and gaps

Despite the growing body of evidence supporting the health-promoting effects of bioactive compounds in fermented foods, several limitations were identified across the included clinical studies.

Although many studies reported statistically significant health outcomes, a considerable proportion did not investigate underlying biological mechanisms. Changes in clinical or biochemical parameters were often not accompanied by biomarker analyses or mechanistic proxies (e.g., AMPK activation, eNOS expression, or cytokine modulation). The absence of mechanistic detail limits causal inference and constrains the interpretation of compound-specific bioactivity beyond correlative associations.

Few studies provided microbial strain-level identification or detailed characterisation of metabolic by-products generated during fermentation. Given the known strain-dependent variability in metabolite synthesis—such as GABA production or peptide release—this lack of specificity limits reproducibility and mechanistic understanding. The absence of this information hinders our ability to link particular microbial species or enzymatic pathways with specific health outcomes.

Many studies failed to quantify bioactive compound concentrations or bioavailability before and after fermentation, or in the final consumed product. Without standardised measurement of key compounds—such as peptides, polyphenols, or isoflavones—it is difficult to establish dose–response relationships, assess bioaccessibility, or evaluate functional potency. This omission weakens the evidence base needed for dietary recommendations and limits product standardisation efforts.

Substantial heterogeneity was observed in study design elements, including sample size, intervention period, placebo composition, and outcome selection. Additionally, variability in fermented food matrices, fermentation parameters, and delivery formats (e.g., beverages, capsules, or whole foods) complicates cross-study comparisons. The lack of standardised reporting frameworks further impedes reproducibility and data integration for meta-analytical purposes.

Most of the evaluated trials were of short duration, limiting the ability to assess the long-term effectiveness and sustainability of the reported health benefits. Furthermore, the absence of multicentre studies restricts the generalisability of findings across populations with diverse dietary habits, genetic backgrounds, and gut microbiota compositions. Addressing this gap is essential to advancing the clinical relevance and global applicability of fermented food interventions.

## 15 Conclusion

This systematic review presents the first comprehensive, compound-centred catalogue of bioactive compounds derived from fermented foods that have demonstrated statistically significant health effects in human observational and intervention studies. By mapping specific compounds to their respective fermented food sources and mechanistic pathways, this work advances our understanding of how fermentation transforms raw substrates into functionally enhanced dietary interventions.

A key strength of this review lies in its systematic selection of human clinical studies in which fermented foods containing specific bioactive compound(s) were directly employed as the intervention or exposure, with clinically relevant outcomes evaluated. By adopting a compound-level analytical framework, this review bridges a critical translational gap between fermentation processes, food science, and human nutrition—offering a more precise and mechanistic understanding of bioactivity than conventional food- or food group-based approaches.

As a result of the findings, future research should prioritise the standardisation of bioactive compound labelling in fermented functional foods, ensuring the quantification and validation of key metabolites. The design and implementation of long-term, multi-centre randomised controlled trials that account for fermentation parameters, microbial strains, and compound stability are equally crucial. Moreover, integrating omics-based mechanistic studies will strengthen causal links between bioactives and clinical outcomes. Finally, the development of regulatory frameworks to support compound-specific health claims will be essential for the translation of scientific evidence into public health policy and functional food innovation.

Fermented foods hold significant promise as vehicles for delivering health-promoting bioactive compounds. Continued multidisciplinary research is essential to harness their full potential in the development of evidence-based dietary strategies for health promotion and disease prevention.

## Data Availability

The original contributions presented in the study are included in the article/Supplementary material, further inquiries can be directed to the corresponding authors.

## Footnotes

^1^ http://osf.io/jn8pf
