# Supplementary material for: Bioactive compounds in fermented foods: a systematic narrative review
Source: Front Nutr. 2025 Jul 2;12:1625816. doi: 10.3389/fnut.2025.1625816 (PMC12282486; doi:10.3389/fnut.2025.1625816)
Supplement: Supplementary file 1 [file Table_1.docx]

**Supplementary Table 1. Bioactive Compounds in Fermented Foods: Health-Promoting Activities, Fermentation Characteristics, Raw Food Sources, and Fermenting Microorganisms Categorised by Health Benefit Domains**

| **Type of Health Benefits** | **Fermented Food** | **Bioactive Compound** | **Health-Promoting Activity** | **Fermentation Conditions** | **Fermentation Type** | **Raw Food Source** | **Fermenting Microorganism(s)** | **Ref.** |
| --- | --- | --- | --- | --- | --- | --- | --- | --- |
| Cardiovascular Health &  Blood Pressure Regulation | Dark Chocolate | Polyphenols (bioactive nitric oxide derivatives) | Antihypertensive, improves endothelial function | Commercially available product | No information | Cocoa | No information | 20 |
|  | Fermented Milk | Tripeptides; Ile-Pro-Pro (IPP) and Val-Pro-Pro (VPP) | Blood pressure-lowering | No information | Lactic acid fermentation | Milk | *L. helveticus* LBK-16H | 10 |
|  | Fermented Milk | IPP and VPP peptides | Antihypertensive, ACE-inhibiting | Fermented for 18–20 h at 37°C until pH 4.0–4.2 | Lactic acid fermentation | Milk | *L. helveticus*, *Lactococcus* sp. | 11 |
|  | Dealcoholized Red Wine | Polyphenols | Blood pressure-lowering, antioxidant | No information | Alcoholic, malolactic fermentation | Red grape | No information | 41 |
|  | Fermented Goat Milk (FGM) | Bioactive peptides | Anti-hypertensive | Commercial product  (chewy tablets) | Lactic acid fermentation | Goat milk | No information | 12 |
|  | Fermented Milk | IPP and VPP peptides | Antihypertensive | Fermented for 22 h at 37°C | Lactic acid fermentation | Milk | *L. helveticus* CM4 | 13 |
|  | Fermented Sour Milk | IPP and VPP peptides | Antihypertensive | Fermented for 24 h at 37°C | Lactic acid, alcoholic fermentation | Skim milk | *L. helveticus, S. cerevisiae* | 14 |
|  | Sour Milk | IPP and VPP peptides | Antihypertensive | No information | Lactic acid fermentation | Sour milk | *L. helveticus* LBK-16 | 15 |
|  | Cocoa drink | Epicatechin | Vascular function | No information | No information | Cocoa | No information | 16 |
|  | Natto | Nattokinase | Blood pressure reduction | No information | Bacterial fermentation | Soybean | *Bacillus subtilis* | 28 |
|  | Miso | ACE inhibitor agents | Antihypertensive | No information | Microbial fermentation | Soybean, Rice | *Aspergillus oryzae* | 29 |
|  | Liquid Yoghurt | IPP and VPP peptides | Antihypertensive | Mixed fermented milk products | Lactic acid fermentation | Reconstituted skim-milk | *L. helveticus, S. cerevisiae, L. delbrueckii, S. thermophilus* | 17 |
|  | Dark Chocolate | Flavanols (epicatechin and procyanidin) | Improves vascular function | No information | No information | Cocoa | No information | 21 |
|  | Fermented Milk | IPP and VPP peptides | Improves vascular function | No information | No information | Milk | *L. helveticus* LBK-16H | 18 |
|  | Liquid Yoghurt | IPP and VPP peptides | Antihypertensive | Mixed fermented milk products | Lactic acid fermentation | Reconstituted skim-milk | *L. helveticus, S. cerevisiae, L. delbrueckii, S. thermophilus* | 19 |
|  |  |  |  |  |  |  |  |  |
| Lipid Metabolism, Cholesterol Homeostasis & Obesity Management | Fermented Orange Juice | Flavonoids | Antioxidant, anti-inflammatory | Fermented for 10 days at 22°C with *Pichia kluyveri* | Alcoholic fermentation | Orange juice | *Pichia kluyveri* | 56 |
|  | Red Date Vinegar | Acetic acid | Anti-hypercholesterolemic | Commercially available product | Acetic acid fermentation | Dates | No information | 53 |
|  | Fermented Orange Juice | Polyphenols (flavonoids), vitamin C | Antioxidant, anti-inflammatory, lipid-lowering | Fermented for 10 days at 22°C with *Pichia kluyveri*, then pasteurized | Alcoholic fermentation | Orange juice | *Pichia kluyveri* | 57 |
|  | Dealcoholized Red Wine | Polyphenols (Pelargonidin, malvidin, delphinidin, gallic acid) | Anti-obesity, cholesterol regulation | No information | Yeast fermentation | Grape | No information | 58 |
|  | Dark Chocolate | Epicatechin, dihydrophenyl derivatives | Gut microbiota modulation, improves HDL levels | No information | No information | Cocoa | No information | 22 |
|  | Doenjang | Polyphenols (Genistein, daidzein), small peptides | Anti-obesity, antioxidant | No information | No information | Soybean | *Bacillus*, *Aspergillus* species | 30 |
|  | Fermented Oat-Based Products | Soluble fibers (β-glucan) | Anticholesterol | Dairy-based: 43°C; Ropy oat: 28°C | Lactic/propionic acid fermentation | Milk, oat-based products | *Lactobacillus* spp., *Streptococcus* spp., *P. damnosus* 2.6 | 66 |
|  | Malleable Protein Matrix (MPM) | Whey proteins | Lipid (triglyceride) -lowering effect | Following fermentation enriched through centrifugation then pasteurization | Lactic acid fermentation | Whey | *Lactobacillus kefiranofaciens* | 68 |
|  | Tempeh Gembus | Dietary Fiber | Lowers LDL-C and total cholesterol | No information | Microbial fermentation | Soy pulp (soybean processing byproduct) | *Rhizopus oligosporus* | 31 |
|  | Kochujang (Soybean-based Red Pepper Paste) | Capsaicin | Anti-obesity | Fermented for 6 months | Microbial fermentation | Red pepper, soybean, rice | No information | 32 |
|  | Kochujang | Capsaicin, daidzein, genistein | Hypocholesterolemic | Fermented for 15–30 days at 30°C | Aerobic fermentation | Soybean-based red pepper paste | *Aspergillus oryzae* | 33 |
|  | Kombucha | Phenolic compounds | Modulates gut microbiota in individuals with obesity | Fermented aerobically for 7 days at 25°C | Mixed fermentation | Black tea leaves | Symbiotic Culture of Bacteria and Yeast (SCOBY) | 74 |
|  | Monascus pilosus Garlic Extract (MGFE) | Monacolin K, Dimerumic acid | Reduces serum lipids and LDL/HDL ratio | Aerobic fermentation with *M. pilosus* and garlic juice for 3 weeks | Single fermentation | Garlic | *Monascus pilosus* | 72 |
|  |  |  |  |  |  |  |  |  |
| Antioxidant Activity & Oxidative Stress Reduction | Chocolate | Epicatechin | Cardioprotective, antioxidant | No information | No information | Cocoa | No information | 23 |
|  | Cocoa Beverage | Epicatechin | Antioxidant activity | No information | No information | Cocoa | No information | 24 |
|  | Fermented Sea Tangle (FST) | γ-aminobutyric acid (GABA) | Enhances antioxidant enzyme activities, reduces oxidative stress | Fermented by *L. brevis* BJ20 for 5 days at 37°C | Lactic acid fermentation | Laminaria japonica (Sea Tangle) | *Lactobacillus brevis* BJ20 | 78 |
|  | Fermented Papaya Preparation (FPP) | Polyphenols | Improves antioxidant status, reduces inflammation and oxidative stress biomarkers | Biofermentation by Osato Research International, Japan | Single fermentation | Papaya | Yeasts | 75 |
|  |  |  |  |  |  |  |  |  |
| Glucose Metabolism & Insulin Sensitivity | Sourdough Bread | Insoluble dietary fibre, organic acids (lactic and acetic acid) | Lower postprandial glycaemic and insulin response | Fermented with *Saccharomyces cerevisiae* and *Saccharomyces exiguous*, along with *Lactobacillus acidophilus* and *Lactobacillus casei* at 28°C for 4-5 hours | Sourdough fermentation | Organic wheat flour | *Saccharomyces cerevisiae, Saccharomyces exiguous, Lactobacillus acidophilus, Lactobacillus casei* | 80 |
|  | Dark Chocolate | Epicatechin, theobromine | Improves glucose metabolism during exercise | No information | No information | Cocoa | No information | 25 |
|  | Rye Bread | Polyphenols | Anti-diabetic effect | Room temperature | Yeast fermentation | Rye | Dry yeast | 85 |
|  | Natto | Polyglutamic acid | Suppresses postprandial blood glucose elevation | No information | Bacterial fermentation | Soybean | *Bacillus subtilis* | 34 |
|  |  |  |  |  |  |  |  |  |
| Hematologic Effects | Dark Chocolate | Epicatechin | Platelet function | No information | No information | Cocoa | No information | 26 |
|  | Dark Chocolate | Flavan-3-ol, catechin | Platelet function | No information | No information | Cocoa | No information | 27 |
|  | Pro-Yogurt (Pro-WPI) | Whey protein-derived bioactive peptides | Improved athletic anaemia | Fermented with *Lactobacillus casei* CNCM-1518 for 1h and then with yoghurt starter for 3.5 h at 38°C | Lactic acid fermentation | Whey protein isolate (WPI) enriched cow milk | *Lactobacillus casei* CNCM 1518 and *Streptococcus thermophilus,*  *Lactobacillus delbrueckii* subsp. *bulgaricus* | 42 |
|  | Natto | Nattokinase | Fibrinolytic/anticoagulant effect | No information | Bacterial fermentation | Soybean | *Bacillus subtilis* | 35 |
|  |  |  |  |  |  |  |  |  |
| Immune Modulation & Inflammation Reduction | Pecorino Cheese | Polyunsaturated fatty acids (CLA) | Immune modulation, body composition regulation | No information | No information | Milk | No information | 100 |
|  | Fermented rice bran  (RBEP) | Arabinoxylan | Immunomodulatory | Fermented with *Lentinus* *edodes* until significant mycelia growth, polysaccharides extracted using alkali extraction and ethanol precipitation | Fungal fermentation | Rice bran | *Lentinus* *edodes* | 105 |
|  |  |  |  |  |  |  |  |  |
| Neuroprotective & Cognitive Health Effects | Non-alcoholic Beer | GABA | Sedative effect | Commercially available product | No information | Non-alcoholic beer (hops) | No information | 111 |
|  |  |  |  |  |  |  |  |  |
| Liver Health & Detoxification | Fermented Turmeric Powder (FTP) | Curcumin | Improves liver enzyme levels (ALT, AST) | Fermented with *Aspergillus oryzae* at 25°C for 36 hours | Single fermentation | Turmeric | *Aspergillus oryzae* | 116 |
|  | Fermented Ginseng Powder (GBCK25) | Ginsenosides (Compound K) | Improves liver function, reduces fatigue | Fermented with *Saccharomyces servazzii* GB-07 and pectinase enzyme | Multiple fermentations | Ginseng | *Saccharomyces servazzii* GB-07 | 118 |
|  |  |  |  |  |  |  |  |  |
| Others | Koji Amazake | Prebiotic components | Improves gastrointestinal health | Commercially available product | Fungal fermentation | Rice | *Aspergillus* oryzae | 121 |
|  | Dietary Vinegar | Acetic acid | Anti-CaOx nephrolithiasis | No information | Acetic fermentation | Grape | No information | 124 |
|  | Rye Bran Bread | Enterolactone, enterodiol, matairesinol | Anti-carcinogenic | No information | No information | Rye bran | Baking yeast | 126 |
|  | Miso | Isoflavones | Reduces sarcopenia in women with T2DM | Fermented with *Aspergillus oryzae* | Mixed fermentation | Soybeans | *Aspergillus oryzae* | 36 |
|  | Fermented Bilberry Extract | Anthocyanins (Cyanidin, Delphinidin, Malvidin, Peonidin, Petunidin) | Improves subjective accommodation, enhances mesopic contrast sensitivity | Yeast-fermented bilberry extract, purified by precipitation with hot alcohol | Alcohol fermentation | Bilberry | *Saccharomyces cerevisiae* | 129 |

**Abbreviations:** MoA, Mechanism of Action; GABA, Gamma-aminobutyric acid; SCFAs, Short-chain fatty acids; HDL, High-density lipoprotein; LDL, Low-density lipoprotein; ACE, Angiotensin-converting enzyme; PPs, Polyphenols; ROS, Reactive oxygen species; NO, Nitric oxide; BP, Blood pressure; PPG, Postprandial glucose; GI, Gastrointestinal; TG, Triglycerides; BMI, Body mass index; CVD, Cardiovascular disease; TC, Total cholesterol; IFN-γ, Interferon-gamma; COX, Cyclooxygenase; PPAR, Peroxisome proliferator-activated receptor; GM, Gut microbiota.
